# The Exhaustion of the Medical Profession

**Published:** 1917-08-18

**Authors:** 


### THE EXHAUSTION OF THE MEDICAL PROFESSION.

Not a few members of the medical profession are concerned with the fate which peace may bring to themselves and their calling. Rumours of political plots and legislative designs to alter the status of medicine in the community have been whispered abroad, and the tyranny of the official bureau is alleged to be imminent. However this may be, it is well to remember that peace has not yet arrived and that the imperious demands of war are far from being satisfied. A recent event suggests that these demands may be matters of considerable concern to the medical profession. This appears in the plain announcement from the Central Medical War Committee that their ability to supply doctors to the Army is practically exhausted. A few appeals have yet to be heard, but the results of these, whatever they be, will not affect the main position, which is that after the end of this month the Committee will no longer be able to satisfy the large claims of the Army Medical Department. Such is the definite announcement sent officially to the Secretary of State for War. Be it remembered that the Committee is in a position to speak with authority. For some two years it has been in the most intimate relation with the demands of the Army on the one hand and with the needs of the civilian population on the other. The position of every medical practitioner in Great Britain has been carefully considered with the object of satisfying these two claims. And now, in the deliberate judgment of the Committee, a point has been reached where no acting civil practitioner can, under existing powers, be transferred from his present duties to service with the Army. In other words, there are no practitioners of military age and fit for service who can with safety to the community be compulsorily conscripted. Over practitioners above military age neither the Committee nor the War Office has at present any legal or compulsory authority.

Plainly something will have to be done, and the choice of method will have important consequences. An obvious step would be to raise the military age for doctors to forty-five years, but it may be doubted whether the profession would suffer this, or, even if they did so, whether the arrangement would secure the numbers which the Army Medical Department claims. An alternative proposal is the mobilisation of the whole profession with power to re-distribute its members, apart from individual wishes, both in military and in civil work. Our own view has already been expressed, and is that if the national cause requires such a step it is justified, even though it is not accompanied by mobilisation of the other members of the community. Schemes to effect this end are believed to be in existence, but what will become of them in the collapse of the National Service Department no man can say. That there will be some opposition to such an enterprise is certain. And there is one essential preliminary. It is that a scrutiny equal in severity to that which has been applied to the civilian profession shall be applied to the large numbers engaged in military service. The profession and the public must be satisfied that the Army is utilising its doctors to the best advantage. The many reports that this is not so cannot be disregarded, and until they have been competently tested legislative authority to conscript civilian doctors will be strenuously contested.

